# Intermittent pacing therapy favorably modulates infarct remodeling

**DOI:** 10.1007/s00395-017-0616-3

**Published:** 2017-04-06

**Authors:** André Uitterdijk, Tirza Springeling, Kevin C. M. Hermans, Daphne Merkus, Vincent J. de Beer, Charlotte Gorsse-Bakker, Eric Mokelke, Evangelos P. Daskalopoulos, Piotr A. Wielopolski, Jack P. M. Cleutjens, W. Matthijs Blankesteijn, Frits W. Prinzen, Willem J. van der Giessen, Robert-Jan M. van Geuns, Dirk J. Duncker

**Affiliations:** 1 Department of Cardiology, Ee-2351, Erasmus MC, University Medical Center Rotterdam, PO Box 2040, 3000 CA Rotterdam, The Netherlands; 2 Department of Radiology, Erasmus MC, Rotterdam, The Netherlands; 3 Department of Pharmacology, CARIM, Maastricht University, Maastricht, The Netherlands; 4 Boston Scientific Corporation, St. Paul, MN USA; 5 Medical Products Division, W.L. Gore and Associates, Flagstaff, AZ USA; 6 Department of Pathology, CARIM, Maastricht University, Maastricht, The Netherlands; 7 Department of Physiology, CARIM, Maastricht University, Maastricht, The Netherlands

**Keywords:** Myocardial infarction, Myofibroblasts, Infarct geometry, Infarct expansion, Infarct healing, Swine

## Abstract

Despite early revascularization, remodeling and dysfunction of the left ventricle (LV) after acute myocardial infarction (AMI) remain important therapeutic targets. Intermittent pacing therapy (IPT) of the LV can limit infarct size, when applied during early reperfusion. However, the effects of IPT on post-AMI LV remodeling and infarct healing are unknown. We therefore investigated the effects of IPT on global LV remodeling and infarct geometry in swine with a 3-day old AMI. For this purpose, fifteen pigs underwent 2 h ligation of the left circumflex coronary artery followed by reperfusion. An epicardial pacing lead was implanted in the peri-infarct zone. After three days, global LV remodeling and infarct geometry were assessed using magnetic resonance imaging (MRI). Animals were stratified into MI control and IPT groups. Thirty-five days post-AMI, follow-up MRI was obtained and myofibroblast content, markers of extracellular matrix (ECM) turnover and Wnt/frizzled signaling in infarct and non-infarct control tissue were studied. Results showed that IPT had no significant effect on global LV remodeling, function or infarct mass, but modulated infarct healing. In MI control pigs, infarct mass reduction was principally due to a 26.2 ± 4.4% reduction in infarct thickness (*P* ≤ 0.05), whereas in IPT pigs it was mainly due to a 35.7 ± 4.5% decrease in the number of infarct segments (*P* ≤ 0.05), with no significant change in infarct thickness. Myofibroblast content of the infarct zone was higher in IPT (10.9 ± 2.1%) compared to MI control (5.4 ± 1.6%; *P* ≤ 0.05). Higher myofibroblast presence did not coincide with alterations in expression of genes involved in ECM turnover or Wnt/frizzled signaling at 5 weeks follow-up. Taken together, IPT limited infarct expansion and altered infarct composition, showing that IPT influences remodeling of the infarct zone, likely by increasing regional myofibroblast content.

**Electronic supplementary material:**

The online version of this article (doi:10.1007/s00395-017-0616-3) contains supplementary material, which is available to authorized users.

## Introduction

The prevalence of heart failure continues to rise worldwide [6, 31]. An important risk factor for heart failure is left-ventricular (LV) remodeling following acute myocardial infarction (AMI), with infarct size as its principal determinant [6, 14, 28]. Despite advances in the treatment of AMI and subsequent cardiac remodeling, a need exists for novel adjunctive therapies [5]. Current strategies under investigation for cardioprotection include pharmacological agents or application of brief mechanical interruptions of coronary blood flow either before ischemia or during early reperfusion to protect the myocardium by reducing infarct size [9, 12]. In addition, evidence from experimental studies shows that intermittent pacing therapy (IPT) is capable of limiting infarct size not only when applied prior to ischemia [21, 46] but also when applied during early reperfusion [1, 3, 45], resulting in a trend towards blunting of subsequent LV remodeling [2]. Importantly, a recent clinical study confirmed pacing therapy to be effective at reducing infarct size in patients, when started at reperfusion [47].

Presently, it remains incompletely understood whether IPT is capable of limiting LV remodeling independent of its protection against acute myocardial necrosis, i.e., when started a few days after reperfusion. Consequently, the aim of the present study was to investigate the effects of dyssynchronous IPT of the left ventricle (IPTVVI), when started in the sub-acute phase after reperfusion, in a large animal model of transmural reperfused AMI produced by a transient coronary artery occlusion (CAO). For this purpose, swine were subjected to a 120-min CAO followed by reperfusion, three days after which they underwent cine-MRI and delayed enhancement MRI (DE-MRI) to assess global LV remodeling and function as well as infarct geometry. Subsequently animals received IPTVVI (3 × 5 min b.i.d.) throughout the five weeks post-AMI.

## Materials and methods

Experiments were performed in thirty-six 5–6-month-old Yorkshire x Landrace pigs of either sex and were conducted in compliance with the “Guide for the Care and use of Laboratory Animals” and after written approval of the Animal Care Committee of the Erasmus MC.

### Acute effects of IPTAAI and IPTVVI on LV function

In four chronically instrumented swine we assessed safety and proper functioning of the pacemakers by investigating the acute hemodynamic responses to intermittent dyssynchronous pacing of the left ventricle (IPTVVI) in comparison with intermittent synchronous atrial pacing (IPTAAI). See Supplemental Methods and Results for detailed protocol and results.

### Effects of chronic IPTVVI on global LV and regional infarct remodeling

#### Surgery

Swine (*n* = 27) were sedated [ketamine 20 mg/kg intramuscular (IM); midazolam, 1 mg/kg, IM; atropine 1 mg, IM], anesthetized [thiopental sodium, 15 mg/kg, intravenous (IV)] and intubated. Anesthesia was maintained with fentanyl (20 µg/kg/h, IV) until LCx occlusion, followed by isoflurane (1–2% v/v). Antibiotic prophylaxis consisted of procaine benzylpenicillin (20 mg/kg, IM) and dihydrostreptomycin (25 mg/kg, IM). Following thoracotomy via the third left intercostal space, a polyvinylchloride catheter was placed in the aortic arch, the pericardium was opened and the proximal LCx was dissected. A unipolar epicardial lead was actively fixed in the anticipated anterior infarct border zone, followed by 2 h of LCx occlusion. The pericardium and chest were closed and the animals were allowed to recover, receiving analgesia (buprenorphine, 0.01 mg/kg/day, IM) for 48 h. Pacemakers were interrogated weekly. Six swine died during the AMI procedure; two swine died during the first 24 h post-AMI; one swine died during transportation to the MRI at 3 days.

#### Experimental protocol

Three days after AMI, cardiac MRI was performed. Swine were sedated and intubated as described above. Mechanical ventilation and peri-imaging breath holds were performed using a mobile ventilator. During MRI, anesthesia was maintained with fentanyl (20 µg/kg/h, IV) and thiopental sodium (100 mg bolus). Muscle relaxation was achieved using pancuronium bromide (2–4 mg, IV). Post-imaging, animals received antibiotic prophylaxis as described above and were allowed to recover. Then, animals were randomized to either MI control or IPTVVI and pacemakers remained switched off (MI control) or were started (IPTVVI). Importantly, none of the remaining 18 swine died prematurely after stratification into MI control (*n* = 11) or IPTVVI (*n* = 7) groups. The IPTVVI pacing protocol was applied every 12 h (6 am and 6 pm), consisting of three times 5-min of pacing at 30 beats/min above sinus rhythm (AV-delay 10 ms) separated by 5-min of normal sinus rhythm. Post hoc analysis of pacemaker readouts could not confirm proper pacing in one IPTVVI animal, whereas in the MI control group two animals received unplanned indiscriminate pacing. Consequently, for final analysis six swine were included in the IPTVVI group and nine swine were included in the MI control group. At one and five weeks post-AMI arterial blood samples were collected in subsets of animals (*n* = 5 per group). Five weeks after onset of treatment, cardiac MRI measurements were repeated and animals were killed for histological and molecular analysis of the infarct zone.

#### Cardiac magnetic resonance imaging

Cardiac MRI was performed and analyzed on a 3T scanner as described before [4, 38]. Global and regional cardiac function and infarct geometry were assessed at baseline (3 days post-AMI), i.e., before treatment and at 5 weeks follow-up (see Supplemental Methods and Results).

#### Biomarkers

Arterial blood samples were collected 1 and 5 weeks post-AMI in EDTA tubes (*n* = 5) and plasma was stored at −80 °C. Markers of inflammation (TNFα) and extracellular matrix turnover (MMP-9, TIMP-1) were quantified using dedicated porcine enzyme-linked immunosorbent assays (ELISA) according to manufacturer’s instructions (see Supplemental Methods and Results).

#### Histology

At 5 weeks follow-up, transverse sections of infarct tissue were fixed in 4% formaldehyde and embedded in paraffin. To quantify myofibroblasts in the infarct area, sections were stained for alpha smooth muscle actin (αSMA; [44]; see Supplemental Methods and Results). Data were expressed as myofibroblast area/total tissue area (%). In addition, lateral wall myocardial tissue of five additional weight-matched healthy swine was quantified for myofibroblast content.

To quantify total collagen content and discriminate collagen fiber type in the infarct area, sections were stained with 0.1% picrosirius red in saturated picric acid (*n* = 6 per group). Collagen content in the infarct region was expressed as stained area/total infarct area (%) using bright field images (four photos per animal). Collagen fiber types were assessed by circular polarizing microscopy in which five polarizing colors were discerned; red (type I collagen, mature), orange, yellow, green, and teal (type III collagen, immature) to discriminate between mature and immature collagen and were expressed as polarized area of interest/total polarized area (%).

#### RT-PCR

Infarct tissue from all animals as well as normal myocardium of the weight-matched non-MI control group (*n* = 5) was homogenized and RNA was isolated. The isolated RNA was assessed for concentration and purity (A260/A280 ratio), and reverse-transcribed into cDNA (see Supplemental Methods and Results). The expression of 21 genes related to myofibroblast presence, regulation or differentiation, were quantified as well as seven genes involved in extracellular matrix turnover (see Table 2 and Supplemental Methods and Results). Quantification of gene expression was performed using the comparative *C*_t_ (Δ*C*_t_) method and results are expressed as ratios to the housekeeping gene cyclophilin.

### Statistical analysis

All data are expressed as mean ± SEM. Data were analyzed using two-way (time × treatment) RM-ANOVA followed by post hoc testing, when appropriate, or within treatment group differences with paired *t* testing, and between treatment group differences with unpaired *t* testing (SPSS 15.0, IBM, Armonk, NY, USA). One-way ANOVA testing with Fisher’s least significant difference correction (LSD) was used to analyze gene expression data. *P* ≤ 0.05 (two-tailed) was considered statistically significant.

## Results

### Acute effects of IPTAAI and IPTVVI on LV function

Safety and proper functioning of ventricular pacing protocols were ascertained in four pigs as evidenced by the absence of ventricular arrhythmias and a programmed increase in heart rate of 30 beats/min (see Supplemental Methods and Results and Supplemental Figure 1 for details).

### Effects of chronic IPTVVI on global LV and regional infarct remodeling

Table 1 summarizes global LV anatomy, function and infarct geometry data at baseline and at follow-up. In MI control animals, two hours of occlusion of the LCx resulted in AMI that comprised 31 ± 2% of the LV. Already at 3-day post-AMI, LV end-diastolic volume (EDV, 80 ± 3 ml) and end-systolic volume (ESV, 54 ± 3 ml) were larger as compared to EDV (68 ± 3 ml) and ESV (30 ± 2 ml) in a historical control group of healthy adolescent swine of similar age [30]. MI resulted in a depressed ejection fraction (EF) of 33 ± 2%, compared to 55 ± 3% in normal healthy swine [30]. During the 5 weeks follow-up, total LV mass did not change, as the increase in mass of the remote myocardium was balanced by an equivalent decrease in infarct mass (Table 1). EDV and ESV further increased to 113 ± 5 and 68 ± 3 ml, respectively, with EF tending to slightly recover from 33 ± 2 to 40 ± 2% (*P* = 0.06). The increase in EDV over time in swine with MI was in part attributable to physiological growth of the heart, as EDV in healthy swine showed a modest increase from 68 ± 3 to 91 ± 6 ml [30]. In contrast to the increase in ESV over time in swine with MI (from 54 ± 3 to 68 ± 3 ml), there was a negligible increase in ESV in healthy swine (from 30 ± 2 to 32 ± 3 ml), associated with an increase in EF from 55 ± 3 to 64 ± 3% [30]. These findings indicate that MI resulted in LV remodeling and dysfunction that was already observed early at 3 days and was sustained throughout the 5 weeks follow-up period.

**Table 1:** Global LV function and infarct geometry

	Post-MI	
	3 days BL	37 days FU
Global LV anatomy and function		
Body weight (kg)		
MI control	29 ± 0	38 ± 1*
IPT	28 ± 1	37 ± 1*
LV mass (g)		
MI control	56 ± 1	58 ± 1
IPT	55 ± 2	55 ± 1
Heart rate (bpm)		
MI control	93 ± 3	80 ± 6
IPT	100 ± 6	78 ± 11
End-diastolic volume (ml)		
MI control	80 ± 3	113 ± 5*
IPT	81 ± 2	110 ± 8*
End-systolic volume (ml)		
MI control	54 ± 3	68 ± 3*
IPT	53 ± 2	67 ± 8
Stroke volume (ml)		
MI control	27 ± 2	45 ± 4*
IPT	28 ± 2	43 ± 3*
Ejection fraction (%)		
MI control	33 ± 2	40 ± 2**
IPT	35 ± 3	40 ± 3*
Infarct geometry		
Remote LV mass (g)		
MI control	39 ± 1	47 ± 1*
IPT	38 ± 1	46 ± 1*
Infarct mass (g)		
MI control	17.6 ± 1.4	11.7 ± 0.8*
IPT	17.1 ± 1.2	9.3 ± 0.9*^,††^
Infarct size (% LV)		
MI control	31 ± 2	20 ± 1*
IPT	31 ± 2	17 ± 2*
Infarct thickness (mm)		
MI control	6.2 ± 0.2	4.5 ± 0.2*
IPT	5.9 ± 0.2	4.9 ± 0.3
Infarct length (#slices)		
MI control	7.8 ± 0.6	8.3 ± 0.6
IPT	8.8 ± 0.3	8.0 ± 0.5*
Infarct length (mm)		
MI control	47 ± 4	50 ± 4
IPT	53 ± 2	48 ± 3*^,†^
Infarct circumference (#segments)		
MI control	13.5 ± 0.8	12.0 ± 1.1
IPT	13.6 ± 1.6	9.4 ± 1.3*^,†^
Infarct circumference (mm)		
MI control	62 ± 3	65 ± 6
IPT	61 ± 5	52 ± 5
Total # infarcted segments		
MI control	73 ± 8	71 ± 9
IPT	90 ± 9	58 ± 8*^,†^

Data are mean ± SEM; MI control, *n* = 9; IPT, *n* = 6

*BL* baseline, *FU* follow-up, *LV* left ventricle

* *P* < 0.05, vs. corresponding BL

** *P* < 0.10, vs. corresponding BL

^†^ *P* < 0.05, ^††^ *P* < 0.10, change by IPT vs. change in control

**Fig. 1:**
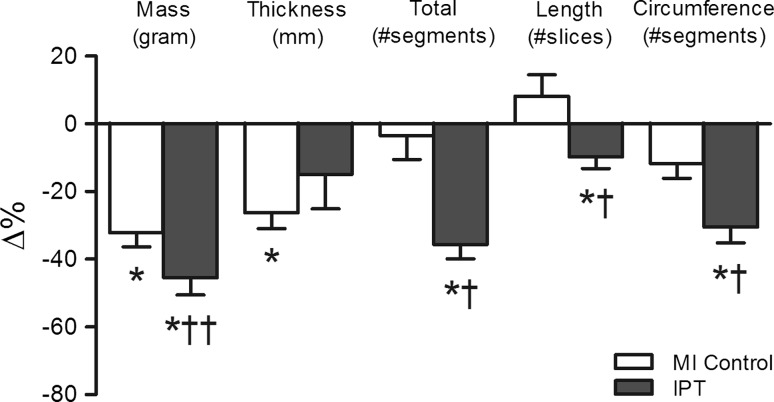
Effect of IPTVVI on infarct geometry. Percent changes in infarct geometry from 3 days baseline values at 5-week follow-up in eight MI control (*white bars*) and six IPTVVI (*gray bars*) swine. Shown are changes in infarct mass, infarct thickness, total number of infarcted segments, number of infarcted slices, and average circumferential infarct length. Data are mean ± SEM; *P ≤ 0.05 vs. corresponding BL; ^†^ *P* ≤ 0.05, ^††^ *P* ≤ 0.10, vs. change in MI control

Global LV and infarct geometry parameters showed no significant differences between IPTVVI and MI control (Table 1) at the 3-day post-AMI baseline. Moreover, the changes in global LV anatomy and function during 5 weeks of follow-up were not different between IPTVVI and MI control. However, infarct geometry responded markedly to IPTVVI (Table 1; Fig. 1). In MI control animals, infarct mass decreased over time as a result of a significant decrease in infarct thickness with no significant changes in infarct length or circumference, and no changes in the total number of infarcted segments. In contrast, in IPTVVI animals, the reduction in infarct mass was primarily due to a decrease in total number of infarcted segments (comprising reductions in both infarct length and circumference), with no significant decrease in infarct thickness. The latter could have played a role in preventing the reduction in infarct mass from reaching statistical significance compared to MI control group (*P* = 0.07; Fig. 1).

#### Infarct composition

**Fig. 2:**
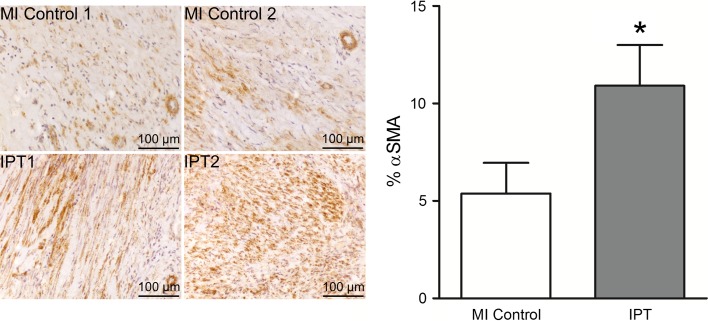
IPTVVI increases myofibroblast presence in the infarct region. *Left panel* αSMA stained infarct tissue of two MI control and two IPTVVI swine (magnification ×20). Brown staining indicates myofibroblasts. *Right panel* percent αSMA-positive cells in infarct tissue from eight MI control (*white bar*) and six IPTVVI (*gray bar*) swine. Data are mean ± SEM; *P ≤ 0.05 vs. MI control

Histological quantification of αSMA positive cells in the infarct region showed a significantly higher amount of myofibroblasts in the infarct area in IPTVVI (10.9 ± 2.1%) than in MI control (5.4 ± 1.6%) swine (Fig. 2). αSMA quantification of referent control tissue (excluding the coronary microvasculature) equaled zero and confirmed the absence of myofibroblasts in healthy myocardial tissue. mRNA-expression data for αSMA between IPTVVI and MI control confirmed the IPTVVI-induced increase in αSMA in histological findings (*P* = 0.04, see Table 2).

**Fig. 3:**
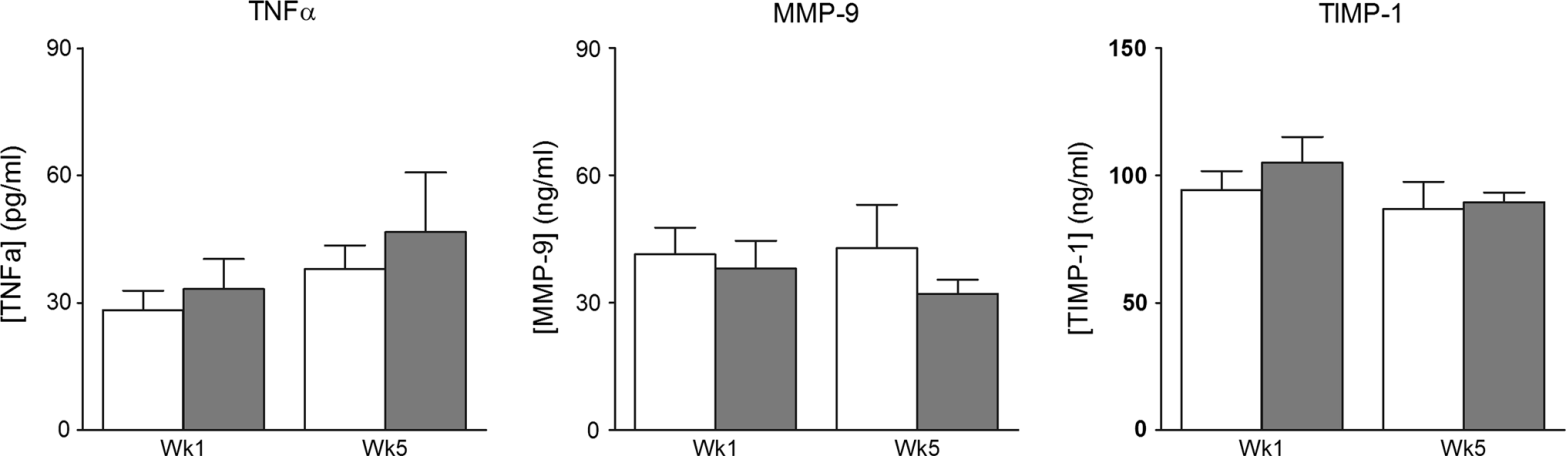
Circulating levels of markers of extracellular matrix and inflammation. Markers for extracellular matrix and inflammation in arterial plasma of five control (*white bars*) and five IPTVVI (*gray bars*) swine. Data are mean ± SEM; *P ≤ 0.05 vs. MI control

Canonical Wnt/frizzled signaling is activated upon injury including myocardial infarction [13, 50]. Indeed, the increased myofibroblast presence in infarcted tissue as compared to non-MI tissue was accompanied by activation of the Wnt/frizzled signaling, as evidenced by increased presence of the Fzd2 receptor and its co-receptor LRP5. However, downstream targets of canonical Wnt/frizzled signaling (axis inhibition protein 2 (Axin), adenomatous polyposis coli (APC) and β-catenin) were not altered in infarcted tissue as compared to non-MI tissue. The inflammatory markers transforming growth factor β1 (TGFβ1) and TGFβ2 were also not altered, while TGFβ3 was increased in MI as compared to non-MI tissue, and its receptors TGFβ1R and TGFβ2R were decreased. Overall, TGFβ-signaling appeared to be reduced in MI as compared to non-MI tissue, as evidenced by a decrease in PAI1. However, IPTVVI had no effect on either Wnt/frizzled or TGFβ-signaling (Table 2), while circulating levels of TNFα were also not different between IPT and MI control animals (Fig. 3).

**Table 2:** Expression of genes related to myofibroblasts and extracellular matrix biology

	Non-MI control	MI control	IPT
Myofibroblast presence, regulation and differentiation			
αSMA	0.5 ± 0.1	0.7 ± 0.1	1.2 ± 0.3*^,†^
Vimentin	2.7 ± 0.6	1.7 ± 0.5	1.6 ± 0.4
Desmin	0.4 ± 0.1	0.4 ± 0.1	0.3 ± 0.1
SPARC	0.5 ± 0.1	1.6 ± 0.5	1.9 ± 0.7**
Tenascin-C	0.1 ± 0.1	1.5 ± 0.9	0.6 ± 0.2*
VEGF-A	1.3 ± 0.4	0.6 ± 0.1**	0.6 ± 0.3**
β-Catenin	0.7 ± 0.3	0.6 ± 0.1	0.4 ± 0.1
TGFβ1	0.8 ± 0.3	0.8 ± 0.1	0.6 ± 0.1
TGFβ2	2.7 ± 1.0	1.8 ± 0.6	1.4 ± 0.1
TGFβ3	0.2 ± 0.1	6.1 ± 2.2**	5.2 ± 1.8
TGFβ1R	3.3 ± 1.3	1.1 ± 0.2*	0.9 ± 0.1*
TGFβ2R	1.7 ± 0.5	0.8 ± 0.1*	0.7 ± 0.1*
Fzd2	0.4 ± 0.1	1.5 ± 0.4**	1.7 ± 0.3*
Fzd4	0.9 ± 0.2	1.3 ± 0.3	0.9 ± 0.2
LRP5	0.2 ± 0.1	0.8 ± 0.2*	0.5 ± 0.2
LRP6	1.1 ± 0.3	0.7 ± 0.1	0.7 ± 0.1
LOX	2.1 ± 0.6	2.0 ± 0.7	3.6 ± 0.7
APC	0.8 ± 0.2	0.8 ± 0.2	0.8 ± 0.1
Axin2	3.1 ± 1.2	3.6 ± 1.2	3.5 ± 1.0
ID1	0.2 ± 0.1	0.6 ± 0.2	0.7 ± 0.2
PAI1	2.6 ± 1.2	1.0 ± 0.2**	0.6 ± 0.2*
Extracellular matrix composition and turnover			
Col1a1	0.9 ± 0.2	2.1 ± 0.7	4.7 ± 1.3*^,†^
Col1a2	2.7 ± 0.7	1.9 ± 0.6	3.5 ± 1.3
Col3a1	0.3 ± 0.1	1.7 ± 0.5**	2.0 ± 0.6*
MMP-2	1.4 ± 0.4	1.7 ± 0.5	2.5 ± 0.7
MMP-9	1.9 ± 0.6	4.5 ± 1.6	4.5 ± 1.5
TIMP-1	0.1 ± 0.1	1.5 ± 0.4	3.4 ± 1.5*^,††^
CTGF	1.6 ± 1.3	1.8 ± 0.5	0.9 ± 0.3

Data are presented as mean ± SEM and expressed as ratios to housekeeping gene cyclophilin; MI control: *n* = 9; IPT: *n* = 6, non-MI control: *n* = 5

*APC* adenomatous polyposis coli, *Axin* axis inhibition protein, *αSMA* alpha smooth muscle actin, *Col* collagen, *CTGF* connective tissue growth factor, *Fzd* frizzled, *ID* inhibitor of DNA binding, *LOX* lysyl oxidase, *LRP* low-density lipoprotein receptor-related protein, *MMP* matrix metalloproteinase, *PAI* plasminogen activator inhibitor, *RT-qPCR* reverse transcriptase quantitative polymerase chain reaction, *SPARC* secreted protein acidic and rich in cysteine, *TGF* transforming growth factor, *TIMP* tissue inhibitor of metalloproteinases, *VEGF-A* vascular endothelial growth factor A

* *P* ≤ 0.05 vs. non-MI control

** *P* ≤ 0.10 vs. non-MI control

^†^ *P* ≤ 0.05 IPT vs. corresponding MI control

^††^ *P* ≤ 0.10 IPT vs. corresponding MI control

Consistent with the role of myofibroblasts in extracellular matrix (ECM) production and turnover, Col1a1-expression at follow-up was significantly increased in IPTVVI animals compared to MI control (*P* = 0.04), while Col3a1 expression was elevated in both MI control and IPT animals (Table 2).

Histological quantification of total collagen content showed no differences between MI control and IPTVVI infarct collagen content at 5 weeks follow-up (73 ± 3 vs. 78 ± 4%, respectively, *P* = 0.34). Furthermore, collagen composition, expressed as the percentage type I collagen fiber (red, mature; 52 ± 7 vs. 65 ± 6%, *P* = 0.22) and type III collagen (teal, immature; 10 ± 2 vs. 7 ± 1%, respectively, *P* = 0.12) were not different between groups (See Supplemental Methods and Results for individual data).

Circulating plasma levels of extracellular matrix turnover (MMP-9 and TIMP-1), measured at 1 and 5 weeks post-AMI, did not differ significantly between treatment groups (Fig. 3), although there was a trend towards an increase in TIMP-1 expression in the infarcted area in IPTVVI vs. MI control (Table 2, *P* = 0.09, two-tailed), that together with the increased Col1a1 expression may reflect increased ECM turnover.

## Discussion

The present study is the first to investigate the effects of dyssynchronous intermittent ventricular pacing (IPTVVI), initiated three days after acute myocardial infarction (AMI) and continued for 5 weeks, on global and regional LV remodeling and infarct geometry in a clinically relevant large animal model. The major findings were that (1) IPTVVI had no effect on global LV remodeling or function, but (2) had a marked effect on infarct remodeling by decreasing the number of infarcted segments without changing infarct thickness. (3) Additionally, IPTVVI increased myofibroblast content in the infarcted area, (4) without changing circulating markers of inflammation and extracellular matrix turnover. These findings provide evidence that intermittent electrical stimulation of the left ventricle may represent a novel means to modulate scar remodeling after MI.

### IPT in AMI and post-AMI remodeling

Several studies in swine [21] and rabbits [45, 46] have shown that pretreatment with ventricular, but not atrial [27], pacing is capable of limiting myocardial infarct size. Subsequent studies have demonstrated that not only preconditioning [21, 45, 46], but also postconditioning with brief periods of ventricular pacing in the early reperfusion phase [1–3, 45] can limit myocardial infarct size. Moreover, the effects of this early protection against myocardial necrosis were sustained over a six week period, resulting in a trend towards blunted LV remodeling and improved LV function [2]. The present study is the first to investigate the effects of prolonged IPT on infarct remodeling, independent of its protection against acute myocardial necrosis in a preclinical animal model. The results of this study clearly demonstrate that IPTVVI started 3 days after reperfusion, at a time when necrosis can no longer be affected, significantly influenced remodeling of the infarct region.

Early studies in humans in the pre-thrombolysis era, reported disproportionate thinning and stretching of the infarcted segment [18, 48, 49]. Recent post-thrombolysis studies in humans with reperfused AMI confirm that regional myocardial wall thinning represents (transmural) myocardial infarction [35]. Furthermore, limited scar burden is associated with improved contractility [35] and blunted remodeling [28], whereas rupture-prone cardiac aneurysms are the consequence of continued ventricular wall thinning [22]. Also, late dilatation of the LV after primary percutaneous intervention remains of clinical significance [37] and may represent a potential target of IPTVVI. Thus, the IPTVVI-induced alteration of infarct geometry may mitigate the sequence of events leading to ventricular wall thinning and limit LV remodeling. Although significant changes in infarct geometry and composition were observed in the present study, these alterations did not yet translate into favorable global LV remodeling at 5 weeks follow-up. This finding, which is similar to previous observations with cell-therapy studies in swine [29], as well as humans [19], with AMI, suggests that more pronounced changes in infarct geometry are required to translate into benefits at the global LV level. This may also explain why a recent clinical study on peri-infarct zone pacing after AMI (PRomPT Trial) reported no difference in LV function or geometry at 18-month follow-up [39]. A limitation of the present study is that we investigated only a single algorithm of pacing therapy, so that we cannot exclude that other algorithms or pacing protocols may produce larger regional effects that do translate into global LV improvements [3]. Clearly, future studies, are necessary to optimize the onset, timing, duration and mode of pacing therapy.

### IPT and infarct geometry: role of myofibroblasts

The infarct zone is increasingly being appreciated as an area with relevant biological activity and therapeutic potential [8, 40]. Cardiac fibroblasts, including the active collagen-secreting myofibroblasts, are the dominant cell type in the infarct region and are recognized as essential in infarct remodeling [7, 20, 41, 43]. Myofibroblasts typically appear in the infarct area at 4–5 days after AMI, reach a peak at 1–2 weeks and continue to reside up to at least 4 weeks [10] and possibly months to years [43]. In the present study, IPTVVI, started at three days post-AMI, increased myofibroblast numbers in the infarct zone significantly. Myofibroblasts could have contributed to the geometric changes in the infarct region produced by IPTVVI in several ways. First, in the infarcted myocardium, myofibroblasts are responsible for collagen turnover thus contributing to the delicate balance between ECM synthesis and degradation [43]. Consistent with a role of myofibroblasts in ECM synthesis, Col1a1 expression was increased by IPTVVI. Moreover, a trend towards increased expression of TIMP-1 was found in infarcts of IPTVVI animals (*P* = 0.09) compared to MI control, which is consistent with the observations of Mukherjee et al. [32] that TIMP-1 co-localizes with myofibroblasts within the infarct zone. Although the increased Col1a1 expression and the trend towards an increase in TIMP-1 did not translate into an increase in collagen as measured with histology, it is plausible that myofibroblasts, a rich source of bioactive molecules [34], modulated infarct geometry by affecting ECM turnover. Second, myofibroblasts are capable of tonic contraction, and could therefore improve structural integrity of the scar and increase mechanical strength in the (sub)acute phase [43]. Particularly, the latter is in concordance with the geometrical changes observed in the present study.

The exact mechanism by which IPTVVI influenced myofibroblast presence was not determined in the present study. However, it is well known that LV pacing results in considerable changes in LV contraction patterns, even in the peri-infarct zone [25, 45], resulting in alterations in regional stretch and loading conditions [17, 33, 36, 46]. Since mechanical tension is an important stimulus for cardiac fibroblast to myofibroblast differentiation [11, 15, 16, 26], it is likely that regional alterations in myocardial stretch produced by IPTVVI stimulated resident fibroblasts to differentiate into myofibroblasts.

No change in circulating arterial plasma levels of the inflammatory marker TNFα was found in IPTVVI vs. MI control swine, at either 1 or 5 weeks after AMI, indicating that the effect of pacing on release of these proteins in pigs with infarcts was not discernible in the systemic circulation even as early as 1 week post-AMI. Hence, altered myofibroblast presence within the infarcted area of IPTVVI treated swine was likely the result of local effects on myofibroblast recruitment and/or differentiation. As myofibroblast migration and differentiation are initiated in the early phase after MI, expression studies at 5-week follow-up can by definition not identify which molecular mechanisms underlie the higher myofibroblast presence. Interestingly, the expression of the Fzd2 receptor, involved in myofibroblast homeostasis [24], was increased in both MI control and IPTVVI treated animals, which may have served to maintain the myofibroblasts in the infarcted region. It has previously been shown that activation of the TGFβ signaling pathway increases myofibroblast presence [23], and therefore it is possible that this pathway was only activated in the early phase of IPTVVI. This is also suggested by a study in swine, in which continuous low dose electrical stimulation within the infarct region, not only resulted in higher numbers of myofibroblasts, but also in elevated TGFβ-R1 activity, when measured as early as one week after onset of stimulation [32]. Expression of TGFβ-R1 and PAI-1, a measure for TGFβ-R1 signaling were downregulated in both the MI control and IPTVVI group as compared to healthy control animals underlining the transient nature of the local inflammatory responses at the molecular level to regional electrical stimulation. Although in agreement with a previous study from our laboratory [42], TGFβ3 was upregulated in the infarcted tissue, this was not altered by IPTVVI, making it unlikely that activation of the TGFβ-pathway at five weeks follow-up contributed to the increased presence of the myofibroblasts in the IPTVVI treated animals.

In conclusion, the present study shows that 5 weeks of IPTVVI, a regionally targeted non-pharmaceutical approach that was safe (no arrhythmias and maintained cardiac output), favorably influenced the infarct remodeling process, likely by increasing myofibroblast content in the infarct region. Thus, whereas MI control swine showed a reduction in infarct mass over the 5-week follow-up period, which was principally due to infarct thinning, IPTVVI resulted in a reduction in infarct mass that was principally due to a decrease in the number of infarcted LV segments while infarct thinning was prevented. Histological assessment revealed increased numbers of myofibroblasts in the infarct zone. Taken together, these findings suggest that IPT in the peri-infarct zone represents a novel adjunctive therapy to favorably modulate infarct healing in patients with acute myocardial infarction.

## Electronic supplementary material

Below is the link to the electronic supplementary material.

Supplementary material 1 (DOCX 977 kb)
