# Supplementary material for: Intermittent pacing therapy favorably modulates infarct remodeling
Source: Basic Res Cardiol. 2017 Apr 6;112(3):28. doi: 10.1007/s00395-017-0616-3 (PMC5383690; doi:10.1007/s00395-017-0616-3)
Supplement: Supplementary file 1 — Supplementary material 1 (DOCX 977 kb) [file 395_2017_616_MOESM1_ESM.docx]

**Intermittent pacing therapy favorably modulates infarct remodeling**

Basic Research in Cardiology 2017

André Uitterdijk, Tirza Springeling, Kevin CM Hermans, Daphne Merkus, Vincent J de Beer, Charlotte Gorsse-Bakker, Eric Mokelke, Evangelos P Daskalopoulos, Piotr A Wielopolski, Jack PM Cleutjens, W. Matthijs Blankesteijn, Frits W Prinzen, Willem J van der Giessen, Robert-Jan M van Geuns, Dirk J Duncker

*Corresponding author:*

Dirk J. Duncker MD, PhD

Dept. of Cardiology, Ee-2351

Erasmus MC, University Medical Center Rotterdam

PO Box 2040,

3000 CA Rotterdam, The Netherlands

Telephone: (+) 31-10-7038066

Telefax: (+) 31-10-7044769

E-mail: d.duncker@erasmusmc.nl

# Supplemental Methods and Results

Acute effects of IPTAAI and IPTVVI on LV function

*Surgery.* Swine were sedated (ketamine 20 mg/kg IM; midazolam, 1 mg/kg, IM; atropine 1 mg, IM), anesthetized (thiopenthal sodium, 15 mg/kg, IV), intubated and ventilated with O_2_/N_2_ (1/3 v/v), to which isoflurane was added (1-2% v/v). Under sterile conditions, the chest was opened and pressure catheters and flow probes were implanted to monitor hemodynamics and LV function [2, 6]. The proximal left circumflex coronary artery (LCx) was ligated to produce AMI [6]. A bipolar atrial lead was sutured onto the right atrium and connected to the subcutaneously implanted pacing device (Insignia Entra 1296, Guidant, St Paul, MN). A unipolar epicardial pacemaker lead was positioned in the mid-anterior wall position near the LAD coronary artery. After proper functioning of the pacing setup was ascertained (Zoom 2920, Guidant, St Paul, MN, United States), the chest was closed and animals were allowed to recover. Swine received analgesia (buprenorphine, 0.01 mg/kg/day, IM) during the first 48h post-surgery.

*Experimental Protocol***.** Five days after surgery, with swine resting quietly, hemodynamic measurements were recorded. Subsequently, swine underwent three consecutive 5-min episodes of AAI or VVI pacing with heart rates at 30 beats/min above resting levels, separated by 5-min intervals of normal sinus rhythm. AAI and VVI pacing protocols were randomly performed on separate days.

*Cardiac MRI and Image analysis*

MRI was performed on a 3.0-Tesla clinical scanner (Signa HD, GE Medical systems, Milwaukee, WI, United States) using a dedicated cardiac four-channel phased array cardiac receiver coil. Repeated breath holds and gating to the electrocardiogram were applied to minimize the influence of cardiac and respiratory motion on data collection. Both baseline and follow-up delayed enhanced MRI (DE-MRI) protocols consisted of cine–MRI and DE-MRI.

Cine-MRI was performed using a steady-state, free-precession technique (FIESTA, GE Medical System). Imaging parameters were: 24 temporal phases per slice, field of view 28-30 x 28-30 cm, matrix size was 128 x 224, repetition time 2.5-2.8 ms, number of average minimal 1.0, time to echo 1.0 ms; flip angle 55 degrees, 12 views per segment, slice thickness was 6.0 mm, slice gap was 0 mm. Using standard techniques to identify the major cardiac axes, two-chamber and four chamber cine-CMR images were obtained. The two- and four chamber end-diastolic images at end expiration provided the reference images to obtain a series of short axis views. This resulted in 8-12 cine breath-hold short-axis images to cover the entire left ventricle. Delayed enhancement imaging was performed with a gated breath hold 2-dimensional T1-inversion recovery gradient-echo sequence minimal of 10 minutes after infusion of Gadoliniumdiethyltriaminepentaacetic acid (0.2 mmol/kg intravenously, Gadovist, Bayern-Schering, Germany). Imaging parameters were; field of view 28-30 x 28-30 cm, matrix size was 192 x 160, repetition time 6.3 ms, number of average minimal 2.0, time to echo 1.6 ms, flip angle 20 degrees, slice thickness was 6.0 mm, slice gap was 0, inversion time 200-300 ms (adjusted to null the signal of the remote myocardium). The slice locations of the delayed enhanced images were copied from the cine-images.

Image Analysis. All images were analysed in a blinded matter using the CAAS-MRV program (version 3.3.1; Pie Medical Imaging, Maastricht, The Netherlands). Cine and delayed enhancement images were acquired during the same imaging session and were matched using identical slice positions [1, 4]

Analysis of all images was achieved by consensus of 2 observers using anatomic landmarks such as papillary muscles and right ventricular insertion sites. The images were analysed using the additional information of the long axis to limit the extent of volume at the base and the apex of the heart [3]. End-diastolic volume (EDV), end-systolic volume (ESV), ejection fraction (EF) and left ventricular mass were measured by manually drawing the endocardial and epicardial contour in end-systolic and end-diastolic phase of the 2- and 4-chamber images with automatic segmentation to the short axis and if necessary corrected manually. Papillary muscles and trabeculations were considered as being part of the blood pool volume. Infarct volume was determined on short axis delayed enhancement images using semi-quantitative analyses for the detection of the delayed enhancement regions (>2SD of the mean SI of the contra-lateral myocardium). The delayed enhancement volume was multiplied by 1.05 g/ml to obtain myocardial infarct mass. For the segmental analyses, only slices with complete circumferential myocardium were used and each slice was divided into 36 segments [4]. Longitudinal infarct length was defined as the total number of slices containing infarction multiplied by slice thickness (6 mm). Mean circumferential infarct length is defined as the average infarct circumferential length of 3 basal slices [3, 4].

*Biomarkers.* Arterial blood samples were collected at 1 and 5 weeks post-infarct in EDTA tubes, centrifuged (10 min at 1460g and 4°C) and plasma was stored within 4h at -80 °C for later analysis of circulating biomarkers. Markers of inflammation (TNFα) and extracellular matrix turnover (MMP-9, TIMP-1) were quantified using dedicated porcine enzyme-linked immunosorbent assays (ELISA) according to manufacturer’s instructions (USCN Life Sciences, Wuhan, China; R&D systems, Minneapolis, MN, USA). Absorbance (450nm) was measured with a SpectraMax M5 plate reader (Molecular Devices Corporation, Menlo Park, CA, USA) and concentrations calculated using a standard curve.

*Histology.* At 5 weeks follow-up, transversal sections of infarct-tissue were fixed in 4% buffered formaldehyde for at least 24 hours and subsequently embedded in paraffin. To distinguish myofibroblasts from fibroblasts in the infarct area, sections of 4 µm were stained for alpha smooth muscle actin [5] (αSMA monoclonal antibody, 1:2000, Sigma, Zwijndrecht, The Netherlands). A minimum of five randomly selected high power fields (90.000 µm^2^ per field) per section were planimetrically quantified in a blinded matter for myofibroblast numbers with vessels excluded (Qwin, Leica, Cambridge, UK). Data were expressed as myofibroblast area / total tissue area (%).

*RT-PCR.* Porcine infarct tissue was homogenized and RNA was isolated using the RNeasy Fibrous Tissue Mini Kit (Qiagen, Hilden, Germany) according to manufacturer’s instructions. The isolated RNA was assessed for concentration and purity (A260/A280 ratio) with a NanoDrop spectrophotometer (Thermo Fischer Scientific, USA). Next, RNA was reverse-transcribed into cDNA, using the iScript cDNA synthesis kit (Bio-Rad, USA). Temperature gradient optimization studies were performed with pooled samples from non-infarct tissue cDNA, and the optimal annealing temperatures for each primer, along with the sequences, are shown in Table 1 of the supplemental data. IQ SYBR Green Supermix (Bio-Rad) was used for the detection of cDNA levels. Quantification of gene expression was performed using the comparative Ct (ΔCt) method and results are expressed as ratios to the housekeeping gene cyclophilin.

# Supplemental Table 1

# Primer sequences and annealing temperatures used in the RT-qPCR studies

| **Sus Scrofa gene** | **Forward primer** | **Reverse primer** | **Annealing Temperature used (^o^C)** |
| --- | --- | --- | --- |
| **Fzd2** | ATAGGCACGTCCTTCCTCCT | GACGGGTGTAGAACTTCCTCC | 62 |
| **Fzd4** | ACATGGGGCATTTCCAGGAG | TACAAGTCGCCTGGGTGAAC | 65 |
| **LRP5** | ACGTGATCGAGTTTGGCCTT | TGTTGTGCATGCAGTCGTTG | 65 |
| **LRP6** | CGTGCCAGTTGGAGGTTTTG | TCCGAAGGCTGTGGATAGGA | 62 |
| **β-Catenin** | ATTGAAGCTGAGGGAGCCAC | ACTCCTAAAGGATGATTTACAGGTC | 62 |
| **TGFβ1** | GTGGAAAGCGGCAACCAAAT | CACTGAGGCGAAAACCCTCT | 65 |
| **TGFβ2** | TGCCTGCGTCCACTTTACAT | AGCTGAGAACCCTGCTATGC | 62 |
| **TGFβ3** | ATGGAGAAGAAACCCAGAGCTT | TCCGACTCGGTGTTTTCCTG | 63.5 |
| **VEGF-Α** | GACCAGAAACCCCACGAAGT | AAATGCTTTCTCCGCTCCGA | 58 |
| **aSMA** | GGACCCTGTGAAGCACCAG | GGGCAACACGAAGCTCATTG | 66.4 |
| **Col1a1** | AGACATCCCACCAGTCACCT | TCACGTCATCGCACAACACA | 62 |
| **Col1a2** | CTTGAGACTCAGCCACCCAG | CCGAATGCAGGTTTCACCAG | 65 |
| **Col3a1** | GCTCCCATCTTGGTCAGTCC | CCATCATTACCTCGAGCCCC | 63.5 |
| **Vimentin** | TCTGGAATCCCTCCCTCTGG | TTGCGCTCCTGAAAAACTGC | 66.4 |
| **Desmin** | GGCTCAGTACGAGACCATCG | GCATCGATCTCGCAGGTGTA | 63 |
| **Tenascin-C** | CACCCCGGTACTTGTTCCAT | CCTCGAAGGTGACAGTTGCT | 57 |
| **SPARC** | ACCCTGTCCAGGTGGAAGTAG | GGCAGAACGACAAACCATCC | 57 |
| **APC** | ACAAAACTGGAAACTGAGGCAT | CGGAGGGACATTTTTGACCG | 63 |
| **LOX** | TCCAAGCTGGCTATTCGACG | AGGATTGTACGGGTCATCGC | 65 |
| **AXIN2** | CAAACCCATGCCTGTCTCCT | CGGAAGAGATAAGCCCCGTC | 65.5 |
| **TIMP-1** | CTGGTCATCAGGGCCAAGTT | GGTCTGTCCACAAGCAGTGA | 63.5 |
| **MMP-2** | GCAGTGATGGCAAGTTGTGG | TTGACATCGTCGTGGGACAG | 65 |
| **MMP-9** | ACTTCGGAAACGCAAAAGGC | AAGAGTCTCTCGCTAGGGCA | 62 |
| **TGFβ1R** | AGATGGGCTCTGCTTTGTCT | CAGATGGTGGCTTTCCTGG | 63 |
| **TGFβ2R** | CTCTGGTGCTCTGGGAGATG | TGCTTTCAACACAGGGATGC | 63 |
| **PAI1** | ATGGCAGCACCGTCTCTGTGC | GAGCTGAGCGTCCAGAATGCTG | 57 |
| **ID1** | CTGGAGTTGGAGCTGAACTCG | GGAACACATGCTGTCTCTGCC | 60 |
| **CTGF** | CACAGAGTGGAGCGCCTGTTC | GATGCACTTTTTGCCCTTCTTAATG | 60 |
| **Cyclophilin** | AGACAGCAGAAAACTTCCGTG | AAGATGCCAGGACCCGTATG | 63.5 |

**Supplemental Results**

Acute effects of IPTAAI and IPTVVI on LV function


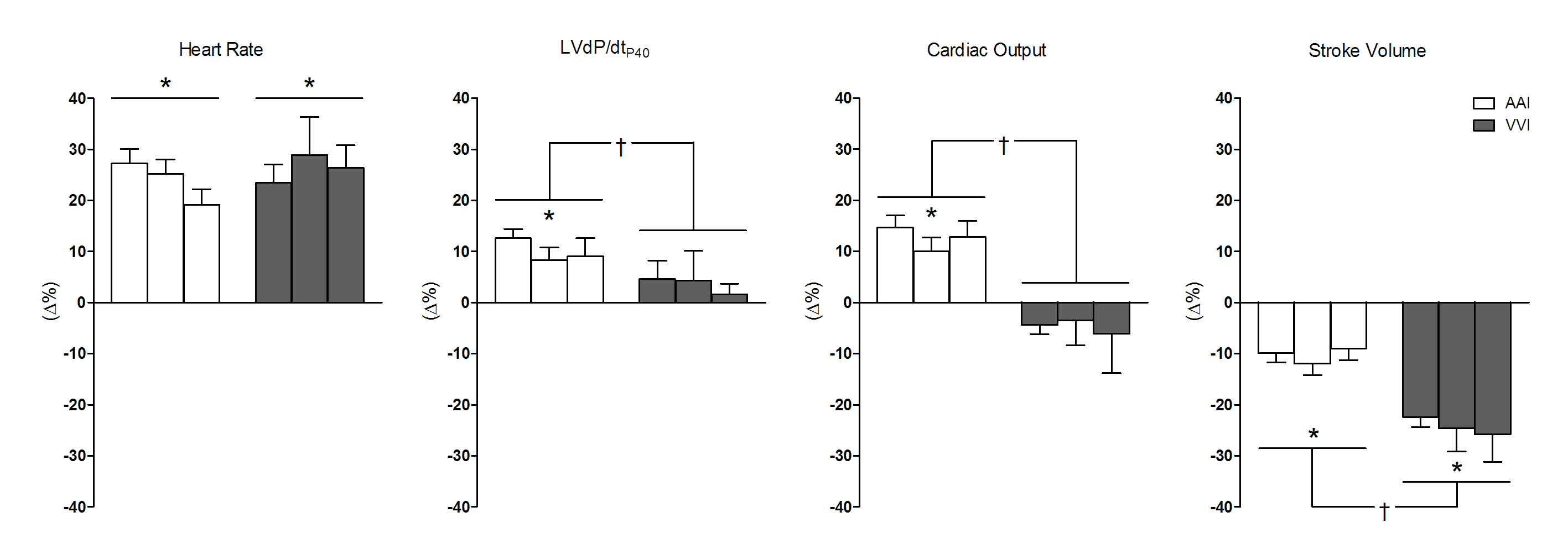
None of the 4 pigs experienced ventricular arrhythmias during AAI or VVI pacing. AAI to 30 beats/min (~25%) above baseline heart rates produced 10-15% increases in global LV contractility (LVdP/dt_P40_) and cardiac output, while stroke volume decreased by 10% likely due to the reduction in LV filling pressure. In contrast, VVI to 30 beats/min above baseline heart rates had no significant effect on LVdP/dt_P40_, and failed to increase cardiac output, as stroke volume decreased by ~25% (Supplemental Figure 1). Importantly, these findings indicated that ventricular pacing was not associated with arrhythmias or hemodynamic instability in swine with a recent AMI.

Supplemental Figure 1 Hemodynamic Effects of Atrial (AAI) versus Ventricular (VVI) Intermittent Pacing

Percent changes from pre-pacing baseline values produced by three consecutive 5-min pacing periods of AAI (white bars) and VVI (grey bars), interspersed by 5-min of normal sinus rhythm. Shown are heart rate, left-ventricular positive dP/dt at a left-ventricular pressure of 40 mm Hg (LVdP/dt_P40_), cardiac output and stroke volume. Data are Mean±SEM; n=4; *P≤0.05 from corresponding BL; †P≤0.05 AAI vs. VVI


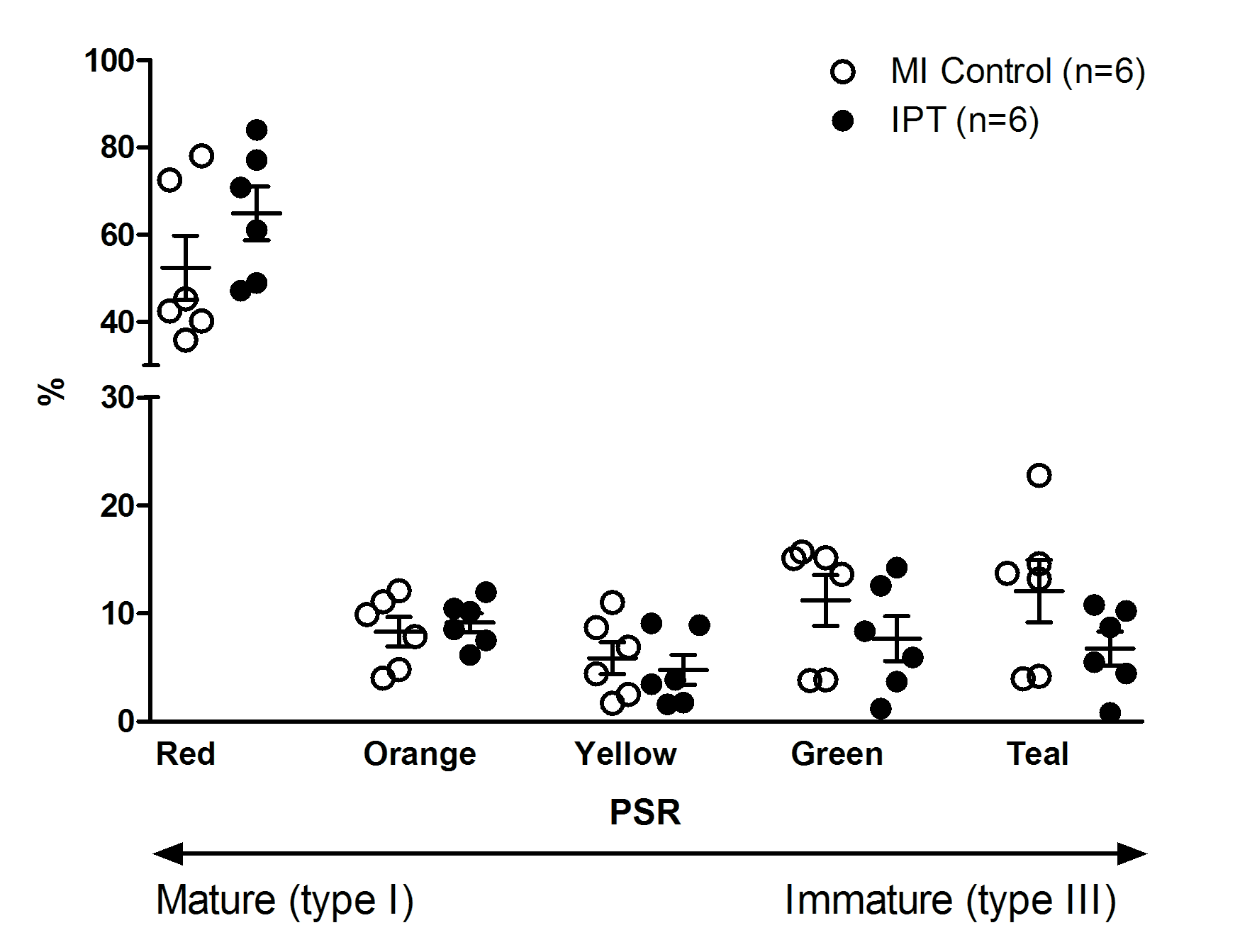


Supplemental Figure 2 Individual picrosirius red histological results of infarct tissue. Results are expressed as polarized area of interest / total polarized area (%). PSR=picro sirius red. Results clearly show that the majority of collagen in infarcts of both groups is mature (tye I).

**References**

1. Baks T, Cademartiri F, Moelker AD, Weustink AC, van Geuns RJ, Mollet NR, Krestin GP, Duncker DJ, de Feyter PJ (2006) Multislice computed tomography and magnetic resonance imaging for the assessment of reperfused acute myocardial infarction. J Am Coll Cardiol 48:144-152. doi:10.1016/j.jacc.2006.02.059

2. Kappers MH, de Beer VJ, Zhou Z, Danser AH, Sleijfer S, Duncker DJ, van den Meiracker AH, Merkus D (2012) Sunitinib-induced systemic vasoconstriction in swine is endothelin mediated and does not involve nitric oxide or oxidative stress. Hypertension 59:151-157. doi:10.1161/HYPERTENSIONAHA.111.182220

3. Kirschbaum SW, Baks T, Gronenschild EH, Aben JP, Weustink AC, Wielopolski PA, Krestin GP, de Feyter PJ, van Geuns RJ (2008) Addition of the long-axis information to short-axis contours reduces interstudy variability of left-ventricular analysis in cardiac magnetic resonance studies. Invest Radiol 43:1-6. doi:10.1097/Rli.0b013e318154b1dc

4. Springeling T, Uitterdijk A, Rossi A, Gorsse-Bakker C, Wielopolski PA, van der Giessen WJ, Krestin GP, de Feyter PJ, Duncker DJ, van Geuns RJ (2013) Evolution of reperfusion post-infarction ventricular remodeling: New MRI insights. Int J Cardiol 169:354-358. doi:10.1016/j.ijcard.2013.09.005

5. van den Borne SW, van de Schans VA, Strzelecka AE, Vervoort-Peters HT, Lijnen PM, Cleutjens JP, Smits JF, Daemen MJ, Janssen BJ, Blankesteijn WM (2009) Mouse strain determines the outcome of wound healing after myocardial infarction. Cardiovasc Res 84:273-282. doi:10.1093/cvr/cvp207

6. van der Velden J, Merkus D, Klarenbeek BR, James AT, Boontje NM, Dekkers DH, Stienen GJ, Lamers JM, Duncker DJ (2004) Alterations in myofilament function contribute to left ventricular dysfunction in pigs early after myocardial infarction. Circ Res 95:e85-95. doi:10.1161/01.RES.0000149531.02904.09
